# Integrated Single‐Cell RNA‐seq and ATAC‐seq Reveals Heterogeneous Differentiation of CD4^+^ Naive T Cell Subsets is Associated with Response to Antidepressant Treatment in Major Depressive Disorder

**DOI:** 10.1002/advs.202308393

**Published:** 2024-06-13

**Authors:** Zuoli Sun, Bowen Zhang, Jingjing Zhou, Yanting Luo, Xuequan Zhu, Yaping Wang, Yi He, Peng Zheng, Ling Zhang, Jian Yang, Gang Wang

**Affiliations:** ^1^ The National Clinical Research Center for Mental Disorders & Beijing Key Laboratory of Mental Disorders Beijing Anding Hospital Capital Medical University Beijing 100088 China; ^2^ College of Life Sciences Beijing Normal University Beijing 100875 China; ^3^ Department of Neurology The First Affiliated Hospital of Chongqing Medical University Chongqing 400016 China; ^4^ NHC Key Laboratory of Diagnosis and Treatment on Brain Functional Diseases The First Affiliated Hospital of Chongqing Medical University Chongqing 400016 China; ^5^ Advanced Innovation Center for Human Brain Protection Capital Medical University Beijing 100069 China

**Keywords:** ATAC sequencing, CD4^+^ naive T cells, major depressive disorder, single‐cell RNA sequencing

## Abstract

The mechanism involved in major depressive disorder (MDD) is well‐studied but the mechanistic origin of the heterogeneous antidepressant effect remains largely unknown. Single‐cell RNA‐sequencing (scRNA‐seq) and assay for transposase‐accessible chromatin using sequencing (ATAC‐seq) on peripheral blood mononuclear cells from 8 healthy individuals and 8 MDD patients before or after 12 weeks of antidepressant treatment is performed. scRNA‐seq analysis reveals a lower proportion of naive T cells, particularly CD4^+^ naive T cells, in MDD patients compared to controls, and in nonresponders versus responders at the baseline. Flow cytometry data analysis of an independent cohort of 35 patients and 40 healthy individuals confirms the findings. Enrichment analysis of differentially expressed genes indicated obvious immune activation in responders. A specific activated CD4^+^ naive T population in responders characterized by enhanced mitogen‐activated protein kinases (MAPK) pathway is identified. E‐twenty six (ETS) is proposed as an upstream regulator of the MAPK pathway and heterogeneous differentiation in activated CD4^+^ naive T population is associated with the response to antidepressant treatment in MDD patients. A distinct immune feature manifested by CD4^+^ naive T cells during antidepressant treatment in MDD is identified. Collectively, this proposes the molecular mechanism that underlies the heterogeneous antidepressant outcomes for MDD.

## Graphical abstract

The mechanisms of depression and heterogeneous antidepressant effect remain unknown. scRNA‐seq and ATAC‐seq on peripheral blood mononuclear cells from healthy individuals and depressive patients is performed. The CD4^+^ naive T cell is an important target for therapeutic heterogeneity the findings. A specific immune activated CD4^+^ naive T subgroup in responders which might be mediated by ETS‐MAPK pathway is revealed.

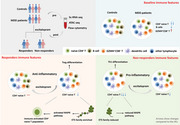

## 1 Introduction

Although the pathogenesis of major depressive disorder (MDD) is not well understood, the hypothesis on immune imbalance has been increasingly accepted in recent years.^[^ ^1^, ^2^ ^]^ There is growing evidence to substantiate an association between MDD and immune cells, a connection that seems to be bidirectional.^[^ ^3^ ^]^ Clinical and animal studies support the relationship between the immune system and depression phenotypes.^[^ ^4^, ^5^, ^6^, ^7^, ^8^ ^]^ As immune cells present heterogeneity in terms of function, many studies now focus on the contribution of immune cell subgroups, especially lymphocyte subsets, to MDD.^[^ ^9^ ^]^

Peripheral blood mononuclear cells (PBMCs), which consist of lymphocytes, natural killer (NK) cells, monocytes and dendritic cells (DC), provide a window to the complexity of the human immune system which can be assessed in the context of both clinical health and pathology. Accumulated experimental animal and human data have highlighted the importance of aberrant PBMCs, especially lymphocytes, in the development of MDD.^[^ ^5^, ^10^, ^11^, ^12^ ^]^ For example, stress is known to suppress immune function, including decreasing leukocyte trafficking, impairing neutrophil phagocytosis, and reducing the number of peripheral lymphocytes.^[^ ^13^, ^14^ ^]^ Flow cytometry (FCM) data analysis showed a reduced percentage of regulatory T (Treg) cells in MDD patients when compared to controls, while the percentage increased after antidepressant treatment.^[^ ^15^ ^]^ Animal studies also demonstrated the association of T cell subsets and depression‐like phenotype.^[^ ^16^, ^17^, ^18^, ^19^ ^]^ However, these studies are usually based on bulk measurements of PBMCs that are noticeably limited by cell‐type heterogeneity.^[^ ^20^, ^21^ ^]^ How the immune system integrates signals and orchestrates responses from different cell types and how it responds to antidepressant drugs are fundamental to our understanding of the immune reaction in depression.

Single‐cell RNA sequencing (scRNA‐seq) may help reveal unique traits of PBMCs in health and disease. Over the past few years, revolutions in scRNA‐seq technology have facilitated unbiased quantification of gene expression in thousands of individual cells, hence, providing a more effective tool for interpreting the role of the immune system in various human diseases. Recently, scRNA‐seq was also used to explore the mechanism involved in neuropsychiatric disorders, such as Alzheimer's disease (AD)^[^ ^22^, ^23^ ^]^ and schizophrenia.^[^ ^24^ ^]^ However, cell‐specific changes to immune cells in MDD patients before and after antidepressant treatment are still unknown.

Here, we performed scRNA‐seq on PBMCs from 8 MDD patients and 8 healthy individuals to obtain an objective transcription‐level atlas for circulating immune cell subsets in MDD patients. We explored the pathophysiology of depression using longitudinal analysis pre‐ and post‐antidepressant treatment derived from the blood transcriptional profiles of individual patients over time. We also performed ATAC‐seq (assay for transposase‐accessible chromatin using sequencing) on the same samples to profile the open‐chromatin regions and define MDD‐associated gene‐regulatory elements at the epigenomic level. To verify the observed changes in immune cell proportions obtained from the scRNA‐seq analysis, we performed FCM on PBMCs obtained from thirty‐five MDD patients and forty healthy individuals. This allowed us to unravel disease‐associated cell‐subpopulation‐specific transcriptome changes and provide robust insights into the cellular heterogeneity of MDD (Figure S1, Supporting Information).

## 2 Results

### 2.1 scRNA‐seq Identified Several Distinct PBMC Clusters and Differentiation Pathways in MDD Patients

**Figure 1:**
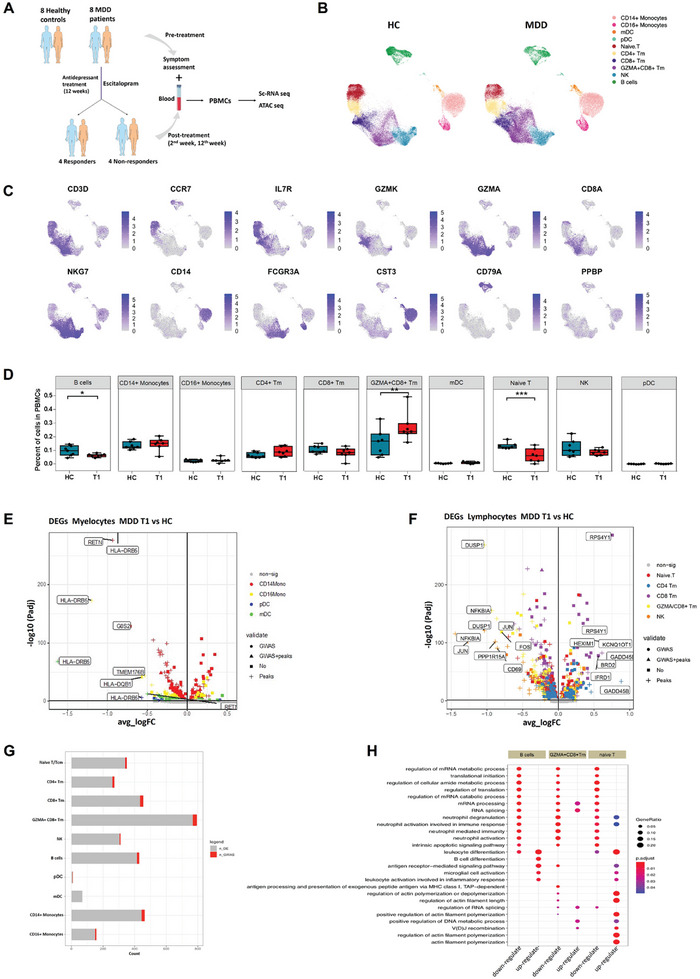
Assessment of major transcriptional profile changes between healthy controls (HCs) and MDD patients at the baseline (before antidepressant treatment). A) A brief experimental flowchart. B) Uniform manifold approximation and projection (UMAP) showing selected marker genes expression derived from scRNA‐seq data; C) UMAPs of MDD patients and HCs, showing the ten main cell types (CD14^+^ monocytes, CD16^+^ monocytes, myeloid dendritic cell (mDC), plasmacytoid dendritic cells (pDC), naive or central memory T cells (naive T), CD4^+^ T memory cells (CD4^+^ Tm), CD8^+^ T memory cells (CD8^+^ Tm), GZMA^+^CD8^+^ Tm, natural killer cells (NK) and B cells). D) Comparison of the proportions of 10 major cell types between MDD patients and HCs. E) Volcano plot showing differential gene expression between myelocytes from HCs and MDD patients before antidepressant treatment. F) Volcano plot showing differential gene expression between lymphocytes from HCs and MDD patients before antidepressant treatment. G) Number of differentially expressed genes in major cell types with red and grey bars indicating genes located within or external to the MDD GWAS risk loci. H, Pathway analysis for differentially expressed genes in naive T, GZMA^+^CD8^+^ T, and B cells.

After completing quality control, 7 MDD patients, comprising 4 responders and 3 nonresponders to treatment, and 7 healthy controls (HCs) were included in the subsequent analysis (**Figure** **1**A; Table S1, Supporting Information). We sequenced 197250 cells at an average of 7045 cells per sample. Ten main clusters were obtained using cluster‐specific genes and the gene expression levels of known markers were determined (Figure 1B,C; Figures S2 & S3, Table S2, Supporting Information). At baseline, B cell and naive T cell proportions were lower, while the proportion of GZMH^+^CD8^+^ Tm cells was higher in MDD patients compared to HCs. No significant differences in other cell‐type clusters were found between the 2 groups. The following data analysis focuses on the 3 changed cell subtypes.

As shown in Figure 1E,F, MDD patients had 349 differentially expressed genes (DEGs) in naive T cells, 794 DEGs in GZMH^+^CD8^+^ T cells, and 429 DEGs in B cells (Table S3, Supporting Information). Gene function enrichment analysis demonstrated a common feature of these cell clusters, which was that genes with up‐regulated expression were enriched for GO terms related to leukocyte differentiation, activation and antigen presentation, or cytoskeleton formation while down‐regulated genes were enriched in functional categories related to neutrophil activation, mRNA and protein synthesis (Figure 1H).

### 2.2 Naive T Cells were Associated with Antidepressant Treatment Outcome

**Figure 2:**
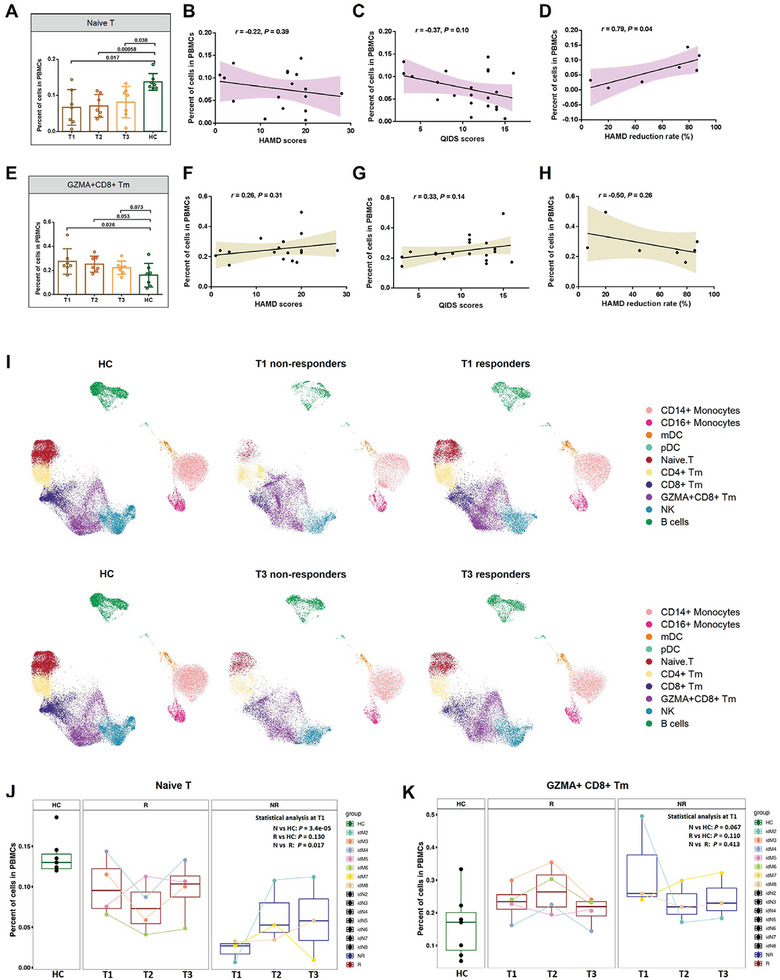
Comparison of major cell types across different cohorts and its relationship with clinical phenotypes. A) A comparison of naive T cells across different conditions. B) Correlation analysis of naive T cells and HAMD‐17 scores in MDD patients. C) Correlation analysis of naive T cells and QIDS scores in MDD patients. D) Correlation analysis of naive T cells and HAMD‐17 reduction rate after antidepressant treatment in MDD patients. E) A comparison of GZMA^+^CD8^+^ Tm cells across different conditions. F) Correlation analysis of GZMA^+^CD8^+^ Tm cells and HAMD‐17 scores in MDD patients. G) Correlation analysis of naive T cells and QIDS scores in MDD patients. H) Correlation analysis of GZMA^+^CD8^+^ Tm cells and HAMD‐17 reduction rate after antidepressant treatment in MDD patients. I) UMAP representation of scRNA‐seq data showing the major cell types across conditions. J) Comparison of naive T cells in healthy and MDD patients with different therapeutic outcomes. K) Comparison of GZMA^+^CD8^+^ Tm cells in healthy and MDD patients with different therapeutic outcomes. Sample time point: T1 = baseline, T2 = 2 weeks, T3 = 12 weeks.

We next examined how the different cellular phenotypes shifted over time following antidepressant treatment. A consistently lower proportion of naive T cells and a higher proportion of GZMH^+^CD8^+^ Tm cells were observed in MDD patients compared to HCs at the baseline or after initiating antidepressant treatment (**Figure** **2**A,E; Figure S4, Supporting Information). The proportion of naive T cells showed a negative trend association with the 17‐item Hamilton Depression Rating Scale (HAMD‐17, r = −0.22, *p *= 0.39) or 16‐item Quick Inventory of Depressive Symptomology–Self‐Report (QIDS, r = −0.37, *p* = 0.10) scores, which was not seen in GZMH^+^CD8^+^ Tm cells (Figure 2B–D,F–H).

To further clarify the relationship between naive T cells and treatment response, patients were divided into responders (4 patients) and nonresponders (3 patients) (Figure 2I; Table S1, Supporting Information). At baseline, the proportion of naive T cells was significantly reduced in nonresponders compared with HCs (*p *= 3.4e–05, Figure 2J), but only showed a decreasing trend in responders (*p* = 0.13). After 12 weeks of antidepressant treatment, neither responders (*P*_2w_ = 0.34; *P*_12w_ = 0.91) nor nonresponders (*P*_2w_ = 0.18; *P*_12w_ = 0.90) showed any significant increase in naive T cells compared with the population at baseline. However, there were no significant differences in GZMH^+^CD8^+^ Tm cells between HCs and responders or non‐responders (Figure 2K).

### 2.3 Subcluster Analysis Reveals CD4^+^ Naive T Cells with Abnormal Metabolic Processes Related to Antidepressant Treatment

**Figure 3:**
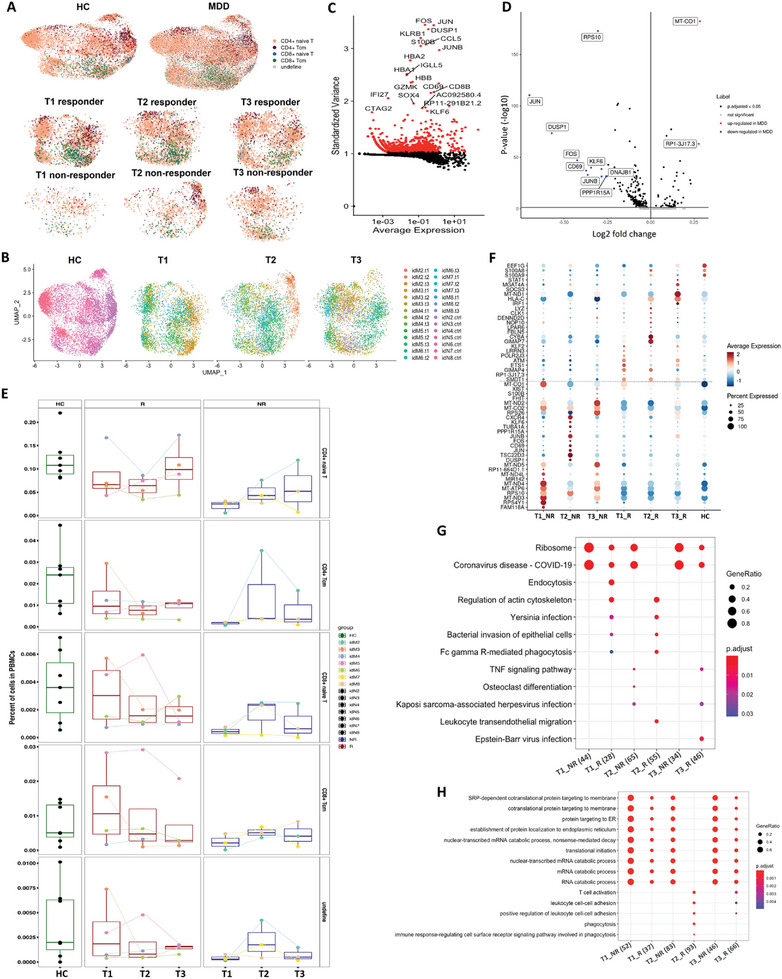
Subcluster analysis to assess major changes in transcriptional profiles of naive T cells between healthy and MDD patients across cohorts. A) UMAP shows the distribution of cell subclusters in participants across different time points. B) UMAP shows the distribution of cell subclusters colored by different individuals. C) Scatter plot showing high variable genes of naive T cell. The top 2000 genes with the highest variation in gene expression were marked in red. D) Volcano plot showing differential gene expression in CD4^+^ naive T cells between HCs and MDD patients before antidepressant treatment. E) Comparison of naive T subclusters in HCs and MDD patients with different time points. F) Top differentially expressed genes in CD4^+^ naive T cells of HCs and MDD patients across conditions. The dotted line separated the up‐regulated expressed genes between responders and non‐responders. G & H) KEGG (G) or GO (H) enrichment pathway or functional analysis of differentially expressed genes in CD4^+^ naive T cells for HCs and MDD patients across conditions.

Subsampling of naive T cells and re‐clustering using the top‐variable features and reference‐based annotation revealed 5 subclusters (**Figure** **3**A,B) with CD4^+^ naive T cells as the main subcluster (Figure 3E). A notable reduction in the proportion of CD4^+^ naive T cells was observed for both responders and non‐responders when compared to HCs at baseline, while the CD4^+^ naive T cells proportion was lower in non‐responders than responders (*p* = 0.0571) or the HCs (*p* = 0.0167). At the end of the 12th week, the proportion of CD4^+^ naive T cells in responders was similar to that in HCs (*p* = 0.649), however, the proportion was still lower in nonresponders compared to HCs (*p *= 0.183). Here we would like to note that the small sample numbers limited the statistical power of our analysis.

A series of DEGs identified in CD4^+^ naive T cells noted that expression of the mitogen‐activated protein kinase (MAPK) pathway‐related genes, such as *JUN, JUNB*, and *FOS*, was significantly reduced in MDD patients compared to HCs (Figure 3D). When responders and non‐responders were compared, we found that DEGs were divided into 2 types (separated by dotted lines in Figure 3F). One type of DEGs was significantly increased in non‐responders whether prior to or postantidepressant treatment (below the dotted line). These genes were mainly associated with mitochondrial energy metabolism and electron transport, such as the *MT‐CO1*, *MT‐CO2*, *MT‐ND2*, *MT‐ND3*, and *MT‐ND4* genes. The other category of DEGs was significantly up‐regulated in responders (above the dotted line) at the baseline. These genes encoded proteins that were mainly responsible for transcriptional regulation to induce the development and differentiation of lymphocytes, such as the *KLF2*, *LRRN3*, *ETS1*, and *GIMAP4* genes. Following antidepressant treatment, the expression of genes encoding functions involved in antigen‐presenting ability, immune response, sterilization, and energy supply such as *CYBA*, *GIMAP7*, *MT‐ND1*, and *HLA‐c* were significantly up‐regulated compared with non‐responders or HCs. Furthermore, the extensive immune response in responders to both pre‐ and post‐antidepressant treatment was also revealed by GO or KEGG enrichment analysis as shown in Figure 3G,H.

### 2.4 Trajectory Analysis Revealed a Specific CD4^+^ Naive T Cells Were Associated with Antidepressant Outcome

**Figure 4:**
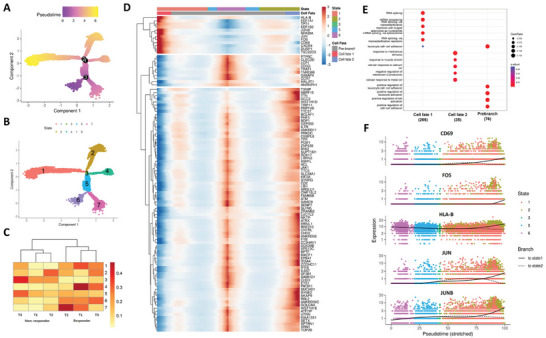
Trajectory analysis of CD4^+^ naive T populations. A) The differentiation trajectory of CD4^+^ naive T cells, coloured‐coded by pseudotime. B) The differentiation trajectory of CD4^+^ naive T cells, coloured‐coded by the states. C) Heatmap showing the proportions of CD4^+^ naive T cells in different states across conditions. D) Heatmap showing different blocks of DEGs in each state of CD4^+^ naive T population. E) Gene set enrichment analysis of CD4^+^ naive T population in each cell fate. F) Scatter plots showing the expression of selected transcription factors in different cell states as the pseudotime progresses.

The above results pointed to the differential activity of CD4^+^ naive T cells between responders and nonresponders. To further understand the immune dynamics, the pseudotime developmental trajectory analysis was carried out with CD4^+^ naive T cells in MDD patients. The CD4^+^ naive T cells mainly clustered into 7 states. We analyzed the data and set cell state 6 as the root of the pseudotime (**Figure** **4**A) according to Figure S5 (Supporting Information), while the states 1 (cell fate 1) and 2 (cell fate 2) were the ending of the pseudotime trajectory. Interestingly, we found the cells with state 1 were more enriched by responders than non‐responders, especially at baseline (before antidepressant treatment) (Figure 4C). Differentially gene analysis and GO enrichment indicated that state 1 cells displayed a clear characteristic with increased expression of immune activation and RNA transcription process, such as HLA‐B, CD69, FOS (Figure 4D–E). Intriguingly, inflammatory regulators in the MAPK pathway, such as JUN, JUNB, and Fos were significantly upregulated in cell fate 1 during pseudotime progression (Figure 4F). Similarly, genes related to CD4^+^ naive T cell activation, including CD69 and HLA‐B, were dominantly expressed in state 1 cells in responders (Figure 4F). As the MAPK pathway played a prominent role in T cell differentiation and inflammation, these results proposed that an immune‐activated naive CD4^+^ T population might dictate the effectiveness of antidepressant treatment.

### 2.5 ATAC‐Seq Identified Distinct Transcription Factor (TF) Binding Motifs

**Figure 5:**
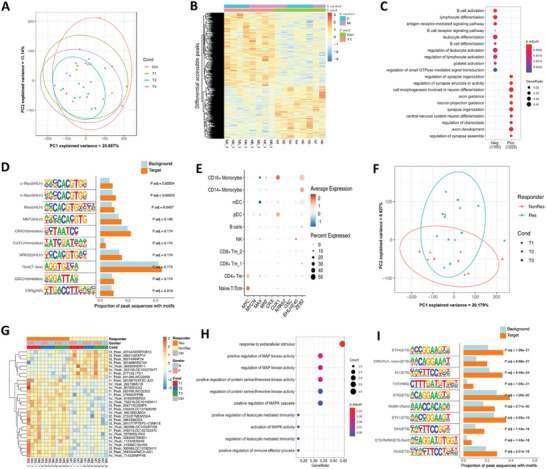
Epigenetically distinct cell subpopulations in MDD patients across conditions. A) Principal Component Analysis (PCA) of ATAC‐seq signals to identify major sources of variation in chromatin accessibility between MDD patients and healthy controls (HCs). B) Hierarchically clustered heatmap of row‐normalized chromatin accessibility in ATAC‐seq for differentially accessible peaks between MDD patients and controls at baseline. C) Pathway enrichment of genes adjacent to differentially accessible peaks between MDD patients and controls at baseline. D) Enrichment of known transcription factor motifs in differentially accessible peaks between MDD patients and controls at baseline. E) Dot plots showing the expression of the top 10 transcription factor motifs in major cell types of MDD patients at baseline from scRNA‐seq data. F, PCA of ATAC‐seq signal to identify major sources of variation in genomics data from patients across disease outcomes. G) Hierarchically clustered heatmap of row‐normalized gene activity in ATAC‐seq for differentially accessible peaks between responders and non‐responders of MDD patients. H, Pathway enrichment of genes flanking differentially accessible peaks for responders and non‐responders. I, Enrichment of known transcription factor motifs in differentially accessible peaks between responders and non‐responders.

We used bulk ATAC‐Seq to profile the open‐chromatin regions and define MDD‐associated gene‐regulatory elements at the epigenomic level (**Figure** **5**). A total of 103968 peaks were obtained from 31 individuals, and 4662 differentially accessible peaks were analyzed (Figure 5B). We noted that genes controlled by down‐regulated accessible peaks in MDD patients were enriched for cell activation and differentiation pathways such as leukocyte activation and differentiation (Figure 5C). Motif analysis (Figure 5D) denoted binding sites for the Myc family of transcription factors such as c‐Myc and n‐Myc. Interestingly, we noted that Myc was mainly expressed in naive T cells (Figure 5E).

Next, we analyzed the differential accessible peaks between responders and nonresponders (Figure 5F,G). We noted that up‐regulation of the MAPK pathway was evident in responders compared to nonresponders at the baseline (Figure 5H). Motif discovery analyses indicated that for the ETS (E26 Transformation Specific) transcription factor family, especially ETV4, its expression was remarkably up‐regulated in responders (Figure 5I; Figure S6, Supporting Information).

### 2.6 FCM Analysis Verified the Changes in Naive T Cells of MDD Patients

**Figure 6:**
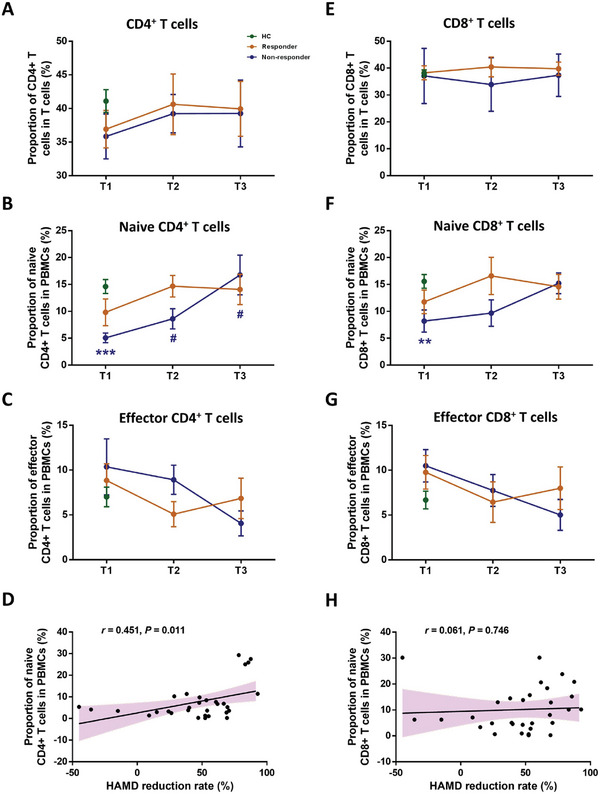
Flow cytometry verification of changes in T cell proportions in MDD patients across conditions. Comparison of major cell types between HCs and MDD patients across conditions are shown in A) (CD4^+^ T cells), B) (naive CD4^+^ T cells), C) (effector CD4^+^ T cells), E) (CD8^+^ T cells), F) (naive CD8^+^ T cells), G) (effector CD8^+^ T cells), respectively. D) Correlation analysis of naive CD4^+^ T cells at baseline and HAMD reduction rate in MDD patients after antidepressant treatment. H) Correlation analysis of naive CD8^+^ T cells at baseline and HAMD reduction rate in MDD patients after antidepressant treatment.

To verify the CD4^+^ naive T cell differentiation in MDD patients prior to or post‐antidepressant treatment, 35 antidepressant‐free MDD patients (19 responders and 16 nonresponders) and 40 HCs were enrolled for the flow cytology study (**Figure** **6**; Figure S7, Table S4, Supporting Information). Consistent with the scRNA‐seq results, the proportion of CD4^+^ naive T cells was reduced in MDD patients at the baseline compared to HCs (*p* < 0.001), and the non‐responders had less CD4^+^ naive T cells than responders at week 0 (*p* < 0.05). Interestingly, escitalopram induced a positive regulation of CD4^+^ naive T cells during the 12‐week antidepressant treatment in non‐responders (*P* < 0.05). CD8^+^ naive T cells also showed changes similar to CD4^+^ naive T cells. Notably, the HAMD‐17 reduction rate only positively correlated with CD4^+^ naive T cells (*p *< 0.05, Figure 6D,H) in patients.

### 2.7 Functional Immune Detection Implicated the Distinct Cytokines Associated with Antidepressant Treatment Outcome

**Figure 7:**
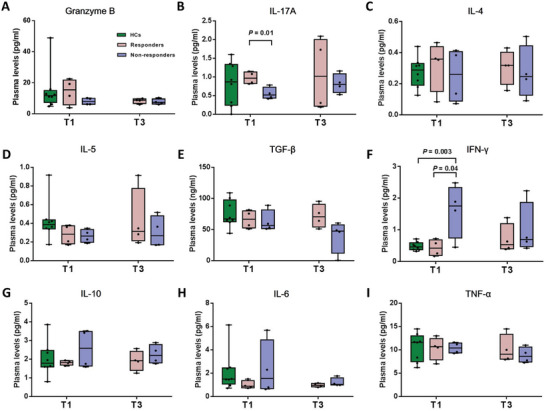
Comparison of cytokines between controls and all MDD patients across conditions.

To identify immune variables that might clarify the functional immune implications of the findings, we measured patient plasma levels of interferon γ (IFN‐γ), interleukin 4 (IL‐4), IL‐5, IL‐17A, tumor necrosis factor α (TNF‐α), Granzyme B, IL‐10, and transforming growth factor β (TGF‐β) (**Figure** **7**). There was a significant difference in IL‐17A levels between responders and non‐responders at the baseline (*p *= 0.01). Furthermore, the nonresponders showed significant increases in IFN‐γ levels compared to the responders (*p *= 0.04) or healthy controls (*p *= 0.003) at baseline.

## 3 Discussion

The aim of this study was to understand the cellular transcriptional changes in MDD patients and their immune response to antidepressant treatment. We noted that the proportion of CD4^+^ naive T cells was significantly decreased in MDD patients compared with that in the HCs. Pseudotime analyses revealed a specific immune‐activated CD4^+^ naive T population, characterized by an enhanced MAPK pathway in responders, for both pre‐ or post‐antidepressant treatment. The subsequent ATAC‐seq data highlighted the importance of the MAPK pathway induced by the ETS family in up‐regulating the immune response and cell differentiation in CD4^+^ naive T cells of responders. FCM revealed the downstream heterogeneous differentiation in CD4^+^ naive T cells, mainly regulatory T cells in responders, as well as Th1 cells in nonresponders (Figure S7). These results revealed the characteristics of immune cells of MDD responders and non‐responders.

We noted a reduction in the number of CD4^+^ naive T cells in patients prior to treatment and the reversal of the initial changes after 12 weeks of antidepressant therapy. There is growing evidence indicating that CD4^+^ but not CD8^+^ T cells are involved in the development of MDD.^[^ ^7^, ^8^, ^25^, ^26^ ^]^ Animal studies also demonstrated that supplementation with CD4^+^ T cells, especially CD4^+^ naive T cells, could significantly reduce behavioral despair in forced swimming tests.^[^ ^7^, ^27^ ^]^ One plausible explanation for the CD4^+^ naive T cells‐mediated depressive phenotype might be the ability of T cells to enter the brain and participate in immunoreactions in the central nervous system (CNS) when the blood‐brain barrier (BBB) is damaged.^[^ ^28^, ^29^ ^]^ T cell migration across the BBB requires the assistance of a variety of adhesion molecules,^[^ ^30^, ^31^ ^]^ which are increased in MDD patients compared to controls.^[^ ^32^, ^33^ ^]^ A second possible reason is CD4^+^ T cell deficiency might contribute to impaired microglia function in the brain. This speculation is supported by a recent study, which showed that the absence of murine CD4^+^ T cells in the brain impaired the maturation of microglia during fetal to adult development and resulted in excess immature neuronal synapses and behavioral abnormalities.^[^ ^34^ ^]^ Third, the decrease in CD4^+^ naive T cells might result in the imbalance of effector T cells, which are activated CD4^+^ T cells derived from CD4^+^ naive T cells.^[^ ^35^, ^36^ ^]^ In this study, the increase in Th1 cells and reduction in Treg cells in MDD patients compared with controls, as captured by FCM, supported this hypothesis (Figure S7, Supporting Information). Interestingly, we found IFN‐γ, which is mainly secreted by Th1 cells, was increased in non‐responders at the baseline. The reduction in CD8^+^ naive T cells in MDD patients compared to HCs might also also be a contributing factor for depression, however, no further analysis was conducted on the CD8^+^ naive T cells in this study. Animal experiments may be able to verify which type of cells are at risk factor for the development of depression. These results suggested that the abnormal differentiation of CD4^+^ naive T cells might perturb the inflammatory response and eventually affect the onset or treatment of depression. It should also be noted that it remains unclear whether the immune changes are the outcome of escitalopram or may reflect “state” changes.

The analysis of DEGs may provide clues on the role and mechanism of CD4^+^ naive T cells in depression. We noted an enhanced immune response not only in CD4^+^ naive T cells but also in other lymphocytes, which might be one of the main causes of depression.^[^ ^3^, ^37^ ^]^ As mentioned previously, there is substantial evidence for an inflammatory protein signature in MDD.^[^ ^1^, ^37^, ^38^ ^]^ Furthermore, the DEGs analysis indicated that this might be due to impaired proliferation and differentiation of CD4^+^ naive T cells. For example, decreased expression of the *MT‐CO1* gene resulted in energy metabolism deficiency while decreased expression of *JUN* and *FOS* genes led to impaired proliferation.^[^ ^39^, ^40^ ^]^ This explanation aligns well with an animal study where the investigators showed that stress‐induced mitochondrial fragmentation in CD4^+^ naive T cells led to excessive production of xanthine into the brain, which resulted in anxiety‐like behavior.^[^ ^7^ ^]^

The responders and non‐responders displayed differential immune and metabolic activity in CD4^+^ naive T cells. The enhanced mitochondrion energy metabolism occurred irrespective of whether a patient receiving antidepressant treatment or not. The excessive mitochondrial activity might result in massive oxygen‐free radical damage, which increases the sensitivity of reactive oxygen species to oxidative stress leading to the disruption of lymphocyte cellular function.^[^ ^41^, ^42^, ^43^ ^]^ Conversely, antioxidant supplementation could exert positive effects on the treatment of depression.^[^ ^44^, ^45^ ^]^ A further explanation is that although the expression of mitochondrial energy‐related genes increased, there was insufficient energy to induce the immune response due to mitochondrial dysfunction in MDD patients. For example, circulating immune cells exhibited the premature aging characteristic of not being able to generate sufficient energy.^[^ ^46^ ^]^ Previous studies have demonstrated mitochondrial fusion dysfunction in CD4^+^ naive T cells in MDD patients or stressed animals.^[^ ^7^, ^47^ ^]^ More importantly, the mitochondrial respiratory chain complex I activity was decreased in responders but increased in non‐responders.^[^ ^48^ ^]^ Taken together, these findings may be useful to form an association between our antidepressant‐induced signatures with cell‐type specific responses in MDD.

The bulk ATAC‐seq data also demonstrated activation of MAPK pathway in responders (Figure 5) similar to the output of the pseudotime analyses (Figure 4). There is reported strong evidence to indicate that activation of the MAPK pathway contributed to an inflammatory reaction as well as lymphocyte proliferation and differentiation.^[^ ^49^, ^50^, ^51^ ^]^ In this study, we found several genes, including *CXCR4*, with increased expression in responders, and the overexpression might induce cell proliferation and differentiation. Silencing the *CXCR4* gene significantly inhibited cell proliferation and promoted cell apoptosis, both of which were proposed to be mediated by the MAPK signaling pathway.^[^ ^52^, ^53^ ^]^ We explored the transcriptional regulation of immune cells and demonstrated that expression of the TF and ETS family was up‐regulated in responders. Interestingly, transcription of the ETS family members, in particular ETS translocation variant 4 (ETV4), is regulated by the extracellular signal‐regulated kinase (ERK) activation.^[^ ^54^, ^55^ ^]^ A recent study indicated that ETV4, in its role as a TF, regulated the hub genes in the brain of a mouse model of depression.^[^ ^56^ ^]^ Although ATAC‐seq was not limited to naive T cells, these results propose that the ETS‐MAPK pathway is required for the maturation and activation of peripheral T cells and may be the upstream regulatory mechanism contributing to the differentially expressed gene profiles between responders and non‐responders.

The main shortcoming of our study is the use of bulk ATAC‐seq and not scATAC‐seq to demonstrate the transcriptional regulation in immune cells. However, we used marker genes that corresponded with the scRNA‐seq data to identify the TF motifs in specific cell clusters, for example, Myc as a transcriptional regulator. The second limitation of this study is that most patients showed moderate depression. It is unclear to what extent these results are translatable to severe depression. Third, the sample size for scRNA‐seq analysis was limited, which affected the statistical power in differential abundance and differential expression analysis.

## 4 Conclusion

In conclusion, this study illustrates key immune cell subsets implicated with depression and suggests that reduction in CD4^+^ naive T cells is a reliable predictor of poor antidepressant treatment efficacy. We also revealed a specific immune‐activated CD4^+^ naive T subgroup in responders and highlighted the activation of these cells might be mediated by the ETS‐MAPK pathway. Our dynamic transcriptome signal analysis demonstrates the functional pathways and complex interactions between immune cell types and provides new insights into the cellular underpinnings of the response to antidepressant therapy.

## 5 Experimental Section

### Human Subjects

All procedures were approved by the Independent Ethics Committee (IEC) of Beijing Anding Hospital (no. 2020–106) and were retrospectively registered in the Chinese Clinical Trial Registry (ChiCTR‐OOC‐17012566) on 4 September 2017. All participants were enrolled between October 2017 and December 2021 and provided written informed consent to participate in the present study. The inclusion criteria for patients were: 1) aged between 18 and 65 years; 2) met the *DSM‐IV* criteria (American Psychiatric Association) according to a clinical interview conducted by experienced research psychiatrists; 3) scored 11 or greater on the QIDS‐SR‐16 as well as scored 14 or greater on the HAMD‐17.^[^ ^57^, ^58^ ^]^ The exclusion criteria included: 1) had a lifetime history of bipolar, schizophrenia, schizoaffective, or other psychiatric disorders; 2) had received antidepressant medication or taken antidepressants for more than 7 days in the past 14 days prior to the current episode; 3) drug or alcohol abuse; 4) pregnancy or breastfeeding; 5) had serious suicide risk (score of ≥ 3 on item 3 of HAMD‐17); 6) had a significant medical illness (e.g., chronic inflammatory disorders, diabetes, cardiovascular disease, thyroid disease, or cancer). The HCs were recruited through advertisements and they were excluded if they had any of the following circumstances: 1) had experienced *DSM‐IV* psychiatric disorder over their lifetime; 2) had significant medical conditions including severe cardiovascular, hepatic, or renal diseases, diabetes, thyroid disease; 3) were pregnancy or breastfeeding.

### Clinical Treatment and Evaluation

After recruitment, patients enrolled for scRNA‐seq received 12 weeks of acute escitalopram antidepressant treatment followed by 12 months of maintenance treatment. Study visits were conducted at the baseline (week 0), weeks 2, 4, 8, and 12 during the acute treatment phase. Patients completed the QIDS‐SR16 and Burden of Side Effects Rating (FIBSER) self‐reporting scales, at week 0 and during each follow‐up visit. The severity of depression was assessed with HAMD‐17 at the start of treatment, and at the 4th, 8th, and 12th week, which was conducted by experienced research psychiatrists in a blind study design. An independent cohort including thirty‐five MDD patients and forty HCs were recruited for the FCM test. The patients received 8–12 weeks of acute treatment for the FCM investigation. Study visits were conducted at weeks 0, 2, 8 /12 during the treatment phase. The severity of depression was assessed with HAMD‐17 at baseline, 2nd, and 8/12th week. For both cohorts, the treatment responders were defined as those who showed a reduction in HAMD‐17 scores above 50% after antidepressant treatment, otherwise, those who did not show a reduction were classified as non‐responders.

### Specimen Collection

Blood was collected at the baseline (t1), 2nd (t2), and 8/12th (t3) weeks after initiating treatment for all patients. PBMCs were collected using a Ficoll‐Paque PLUS density gradient media (Solarbio Life Science, Beijing, China), and kept frozen in CryoStor (Stem cell technologies, Cat# 0 7930) at a final concentration of ≈1 × 10^5^ live cells mL^−1^. The entire PBMCs isolation process was completed within 3 h. Cell suspensions were stored in liquid nitrogen until scRNA‐seq and ATAC‐seq were performed.

### scRNA‐seq

scRNA‐seq and immune profiling were performed using the 10X Genomics Chromium Single Cell platform. After quality control, LogNormalization (Seurat function) to each cell was applied, where original gene counts were normalized by total UMI (Unique Molecular Identifier) counts, multiplied by 10000 (TP10K), and then log10 transformed (TP10k+1). Next, the data regression for total UMI counts was scaled and performed Principal Component Analysis (PCA) based on the 2000 most variable features identified using the “VST” (variance stabilizing transformation) method implemented in Seurat. Subsequently, the cells were clustered using the Louvain hierarchical clustering algorithm based on the top 15 principal components with a resolution of 0.5. For visualization, Uniform Manifold Approximation and Projection (UMAP) based on the top 15 principal components was applied.

### Data Processing

To annotate a meaningful biological cell identity for each cluster, the double‐checking strategy for the inference was followed. Specifically, differential expression testing was performed between cells in 1 cluster and all other cells in the dataset were used to identify data‐derived marker genes. Up‐regulated genes from the cluster of interest were ranked by the Wilcoxon rank‐sum test and compared with PBMC markers reported in the literature. Then, the results were summarized from the comparison of data‐derived marker genes with public databases and direct visualization of the expression pattern of literature‐derived marker genes. Finally, expression levels in each of the identified cell clusters to manually check the cell identities were visualized (Figure S2, Table S2, Supporting Information). Cell clusters with an aberrant number of mitochondrial genes or mixed lymphocyte and myelocyte markers were classified as contaminants and removed from further analyses. As NK cells share many transcription‐level markers with cytotoxic T cells, the NK cluster was annotated based on cells lacking expression of CD3 (CD3D, CD3E, CD3G) and nonzero expression of CD16 (FCGR3A) and NKG7. Specifically, for naive T cell sub clustering, the reference‐based automatic annotation with preinstalled BlueprintEncodeData reference datasets in SingleR (https://rdrr.io/github/dviraran/SingleR/) to obtain a more detailed identification of CD4^+^ or CD8^+^ naive and central memory T cells was applied.

### Data Analysis

To test the differences in cell composition between MDD patients and healthy individuals in the single‐cell RNA‐seq datasets, the Dirichlet‐multinomial regression, which takes compositional dependencies into account, to the proportion of cell types in each sample as suggested by a previous study was applied. To confirm the results and for comparison across responders and non‐responders from different time points, where only limited sample sizes could be used, the Wilcoxon signed‐rank test was applied.

### ATAC‐seq

To prepare the library for ATAC‐sequencing, frozen PBMCs from each donor were thawed and resuspended at 37 °C. The supernatant was removed, and each pellet was carefully resuspended in the transposase reaction mix for 30 min at 37 °C. The purified DNA was eluted by adding 12 µL of Elution Buffer to the eluting membrane, letting it stand for 1 min at 37 °C, and then centrifuging at 10 000 *g* for 1 min. The sample libraries were then stored at −20 °C until further processing. Finally, the ATAC‐seq libraries were loaded on the BGISEQ‐500 ATAC‐seq platform for 50 bp paired‐end sequencing, where each sample was sequenced to at least 110 million reads (performed by BGI, Shenzhen).

### Data Analysis

ATAC‐seq data was processed based on the ENCODE ATAC‐seq pipeline (v1.8.0) https://www.encodeproject.org/atac‐seq/. Briefly, reads were first trimmed by Cutadapt (v2.5) to remove Nextera sequencing adapters as well as low‐quality bases. Then, the trimmed reads were aligned to the ENCODE reference genome (hg38) with bowtie2 (v2.3.4.3). Postalignment quality control was performed with Picard (https://broadinstitute.github.io/picard/) and SAMtools (v1.9) to filter out duplicated unmapped and multi‐mapped reads or reads with MAPping Quality (MAPQ) values of < 30. Next, for each sample, MACS2 (Model‐based Analysis of ChIP‐seq) (v2.2.4) was used for peak calling, then peaks were filtered and merged into one optimal peak set according to ENCODE ATAC‐seq standards (https://www.encodeproject.org/atac‐seq/).

### Quality Control

Differential peak analysis was performed based on this optimal peak set, adapted from RNA‐seq differential expression detection methods. HOMER (v4.11.1) (http://homer.ucsd.edu/homer/) was used to annotate these peaks into regulatory elements, transcriptional factor motifs, and their nearest genes. For quality control, optimal peaks generated by HTSeq (v0.12.4) with less than 150 reads mapped across all samples were removed from the analyses. Then, the filtered read count matrix for these optimal peaks was loaded into the R DESeq2 package (https://bioconductor.org/packages/devel/bioc/vignettes/DESeq2/inst/doc/DESeq2.html) for differential accessible peak analysis. Peaks with a Benjamini and Hochberg adjusted *p‐*value < 0.1 and log2‐Fold‐Change > 0.5 were regarded as significantly differentially accessible peaks, while peaks with raw *p‐*value < 0.05 and log2‐Fold‐Change > 0.5 were regarded as nominal differentially accessible peaks. R ggplot2/p heatmap packages were used for visualization, and the accessibilities of each peak were centered to ‘0′ in the heatmap.

### Flow Cytometric Analyses

FCM analysis was used to determine the proportions of immune cells in thirty‐five MDD patients and forty healthy individuals to verify the scRNA‐seq results. All antibodies were purchased from BD Biosciences (CA, USA). The PBMC suspensions were collected from the heparin‐anticoagulated whole blood of the subjects and then split into 2 different panels to identify lymphocyte subsets. For intracellular staining with FoxP3, cells were first stained with CD3‐BV786 (BD Biosciences Cat# 563 800), CD4‐BB700 (Cat# 566 392), CD8‐BV711 (Cat# 563 677), CD19‐BV510 (Cat# 562 947), CD56‐BV650 (Cat# 564 057), CD16‐PE‐CF594 (Cat# 562 293), CD25‐BB515 (Cat# 564 467), CD127‐BUV737 (Cat# 612 794), CD45RA‐BV421 (Cat# 562 885), CCR6‐BV605 (Cat# 562 724), CCR7‐PE‐Cy7 (Cat# 557 648) and CXCR3‐BUV395 (Cat# 565 223) monoclonal antibodies and BD Horizon Fixable Viability Stain 780 (FVS780, Cat# 565 388) at 4 °C for 30 min. After centrifuging at 400 *g* for 10 min, the cells were permeabilized and fixed using fix/perm (Cat# 00‐5523‐00, eBioscience, California, USA) for 30 min. After centrifuging at 400 *g* for 5 min, the cells were stained with FoxP3‐APC‐E7 (Cat# 17‐4777‐42, eBioscience, California, USA) antibody at 4 °C for 30 min. Then the cells were washed with 1 mL Phosphate‐Buffered Saline (PBS) 3 times and loaded on the BD FACSCanto II. For the detection of Th17 cells, the PBMC suspensions were stimulated with a leukocyte activation cocktail with BD GolgiPlug (Cat# 550 583) for 4 h at 37 °C under a 5% CO_2_ environment. After centrifuging at 400 *g* for 10 min, the cells were stained with CD3‐BV786, CD4‐BB700, and CD8‐BV711 monoclonal antibodies and FVS780 at 4 °C for 30 min. After centrifugation at 400 *g* for 10 min, the cells were permeabilized and fixed using intracellular fixation and permeabilization buffer (Cat# 88‐8823‐88, eBioscience, California, USA) for 35 min, and stained with Anti‐Interleukin‐17A (IL‐17A) antibody at 4 °C for 30 min. Then the cells were washed with 1 mL PBS, loaded on the BD FACSCanto II, and analyzed with the BD FACSDiva software.

### Cytokines Measurement

Plasma from all patients was harvested at the baseline (t1), 2nd (t2), and 8/12th (t3) weeks after initiating treatment and at the baseline from healthy controls. Circulating cytokines were quantified using the automated ultrasensitive Ella Simple Plex assay technology (ELLA microfluidic analyzer, Protein Simple; Bio‐techne, San José, CA, USA). Three kinds of Simple Plex cartridge (Cat# SPCKB‐PS‐001369, #SPCKC‐PS‐00006252, #SPCKC‐PS‐003199) were used to the measurement of IFN‐γ, IL‐4, IL‐5, IL‐6, IL‐17A, TNF‐α, Granzyme B, IL‐10 and TGF‐β concentrations. The samples were measured in triplicate, and the average values were used to calculation. Analysts were blinded for subjects’ clinical status.

### Statistical Analysis

For the differential expression analysis in PBMCs, hypergeometric tests using gene lists from MSigDB v.6.2 were performed and corrected for multiple‐hypothesis testing using the False Discovery Rate procedure. DEGs between the cell types as well as between different sample groups were identified using a two‐sided Wilcoxon rank‐sum test. A *p*‐value adjusted based on a Bonferroni correction of less than 0.05, was considered statistically significant. The Mann‐Whitney U test was used to compare the cell proportions between groups. In the FCM dataset, the independent *t*‐test or Mann‐Whitney U test was used to assess the differences in proportions of cell types between MDD patients and HCs, or between responders and non‐responders. Changes in cell proportions at the baseline, 2nd, and 12th weeks were analyzed via paired *t*‐test. Partial correlation analysis was used to assess the relationship between cell proportions and depression severity in MDD patients from baseline to endpoint with age, gender, and BMI as covariates. A *p‐*value less than 0.05 was considered statistically significant.

## Conflict of Interest

The authors declare no conflict of interest.

## Author Contributions

Z.S., B.Z., and J.Z. contributed equally to this work. J.Y. and G.W. are co‐senior authors. G.W., J.Y., and Z.S. obtained funding for this study. J.Z, X.Z, and L.Z. recruited the patients and collected the samples. Z.S., Y.W., and Y.H. performed the experiments. B.Z. analyzed the data, and Y.L. provided technical support for data analysis. Z.S. and B.Z. wrote the manuscript. G.W., J.Y., and P.Z. were responsible for the study design, results discussion, and manuscript preparation for publication.

## Supporting information

Supporting Information

Supporting Information

Supporting Information

## Data Availability

The data that support the findings of this study are available in the supplementary material of this article.
