# Supplementary material for: Integrated Single‐Cell RNA‐seq and ATAC‐seq Reveals Heterogeneous Differentiation of CD4+ Naive T Cell Subsets is Associated with Response to Antidepressant Treatment in Major Depressive Disorder
Source: Adv Sci (Weinh). 2024 Jun 13;11(30):2308393. doi: 10.1002/advs.202308393 (PMC11321657; doi:10.1002/advs.202308393)
Supplement: Supplementary file 1 — Supporting Information [file ADVS-11-2308393-s003.docx]

**Supplementary materials**

**Supplementary Figures**

Figure S1. Experimental workflow.

Figure S2. UMAP of scRNA-seq data showing the ten main clusters across samples (A) or conditions (B).

Figure S3. Top expressed genes of each major cell types in both MDD patients and healthy controls derived from scRNA-seq data.

Figure S4. Comparison of cell subtypes proportions across conditions.

Figure S5. The marker genes of states in CD4^+^ naive T cells (A). The differentiation trajectory of CD4^+^ naive T cells, coloured-coded across conditions (B).

Figure S6. Enrichment of known transcription factor motifs in differentially accessible peaks between MDD patients and controls.

Figure S7. FACS comparison of helper T cells (Th) and regulatory T cells (Treg) between healthy and MDD patients with different therapeutic outcomes.

**[Figures below]**


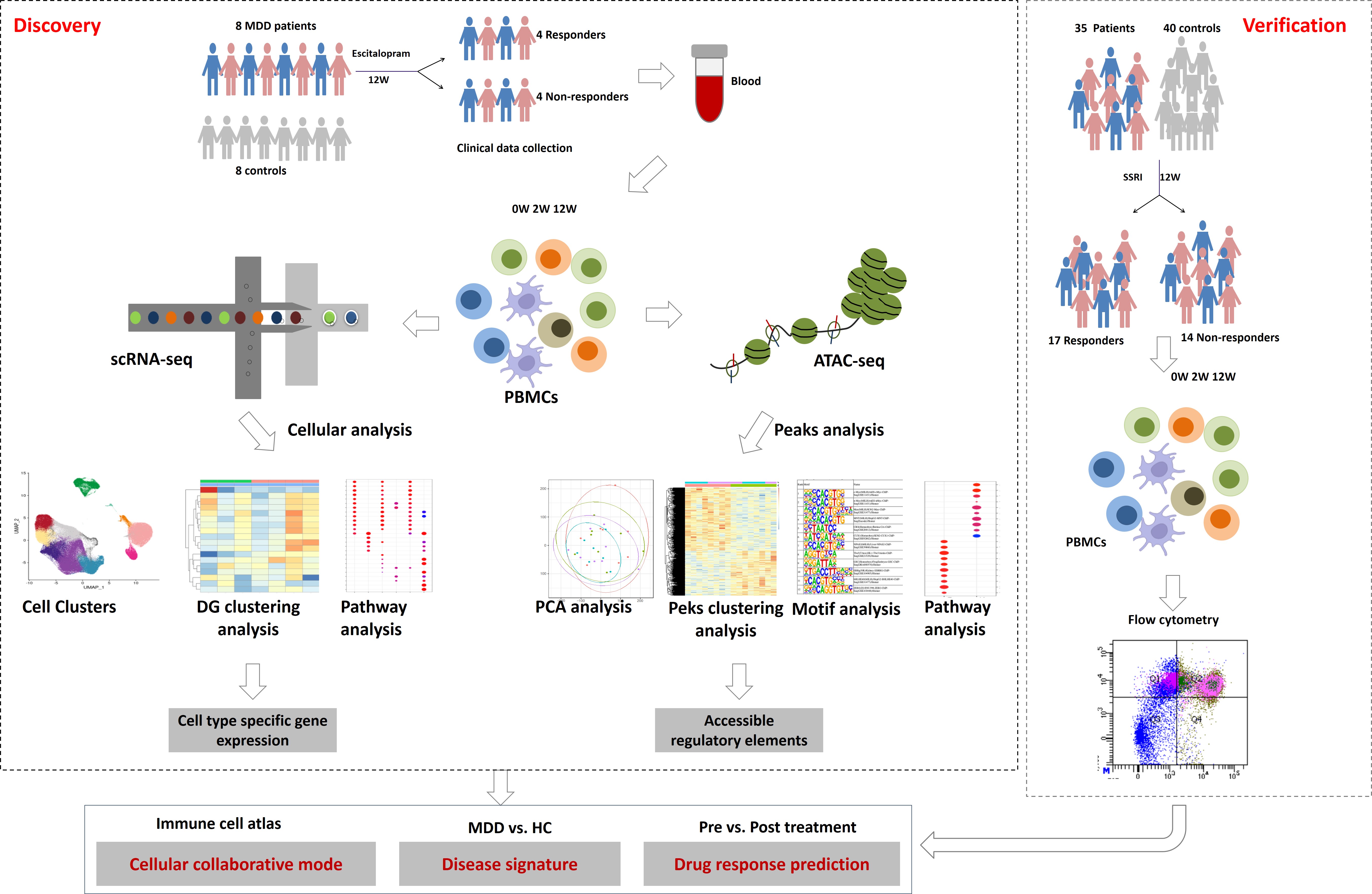


**Figure S1. Experimental workflow.** Briefly, PBMCs were isolated from eight healthy controls and eight MDD patients prior to and post 12 weeks escitalopram antidepressant treatment. The scRNA-seq and bulk ATAC-seq approaches were used to explore the transcriptional characteristics at single cell level, followed by flow cytometry in an independent cohort of patients and controls to verify the scRNA-seq analysis.


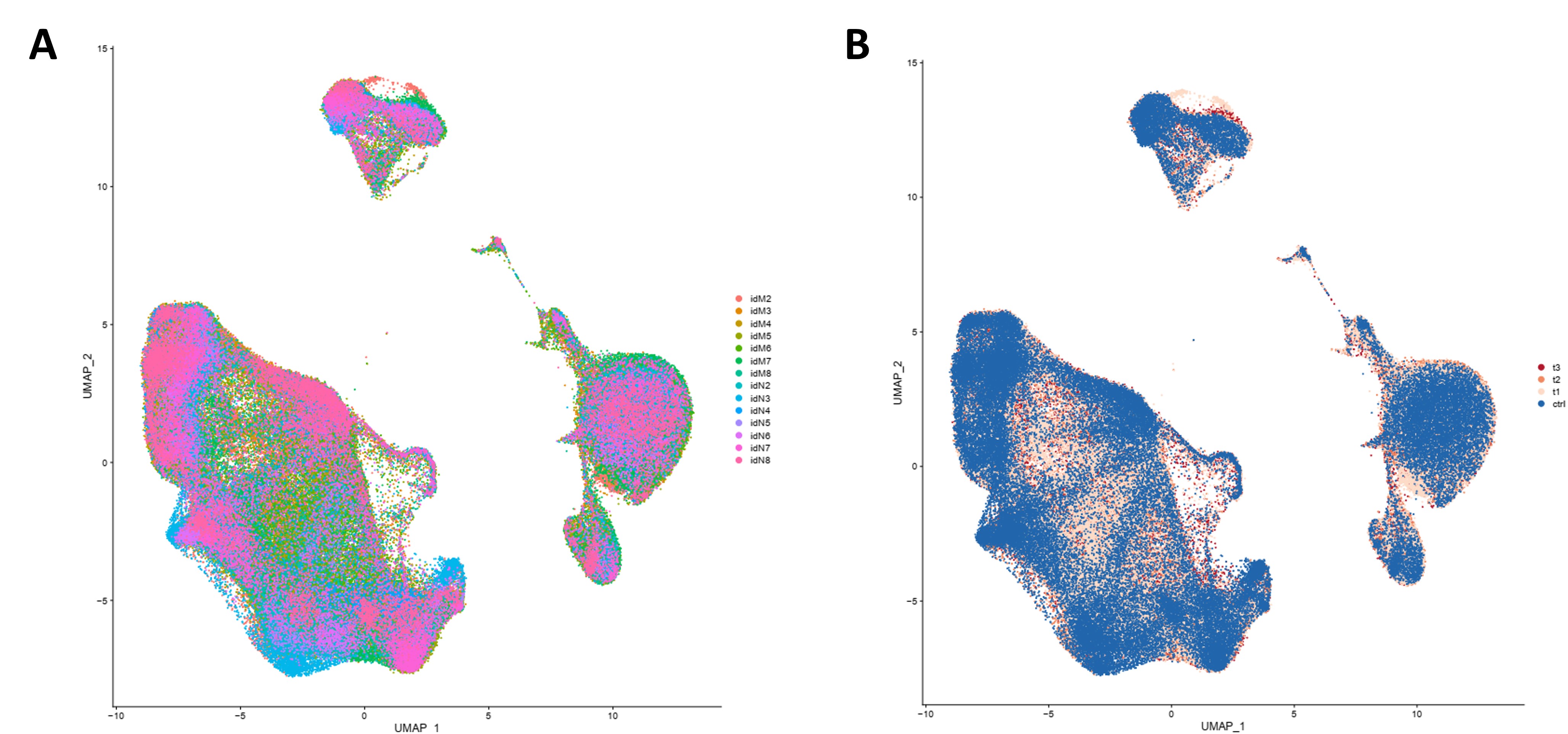


**Figure S2. UMAP of scRNA-seq data showing the ten main clusters across samples or conditions.** A, UMAP of the ten main clusters across samples at baseline for seven HCs and seven MDD patients. The colors indicate the legend refer to the individuals in this study. M2 to M8 represent the MDD patients, while N2 to N8 represent the healthy participants. B, UMAP of the ten main clusters in all subjects, across conditions, including 0^th^ week, 2^th^ week and 12^th^ week.


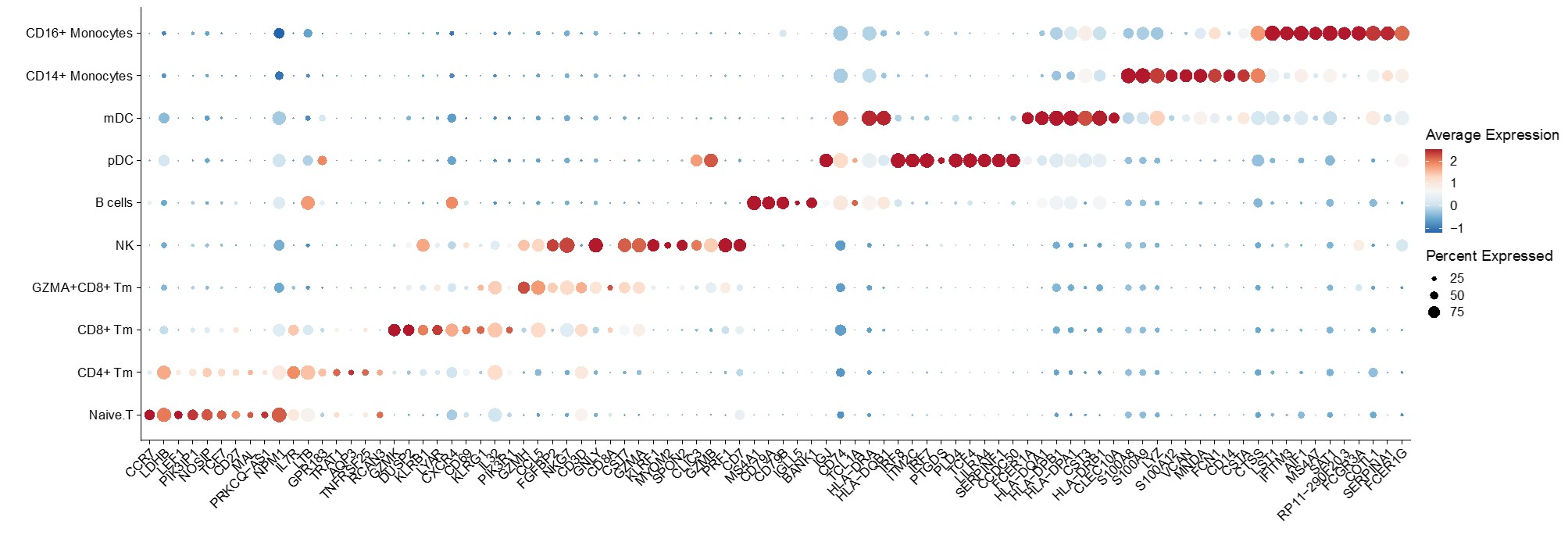


**Figure S3. Top expressed genes of each major cell types in both MDD patients and Healthy controls derived from scRNA-seq data.**


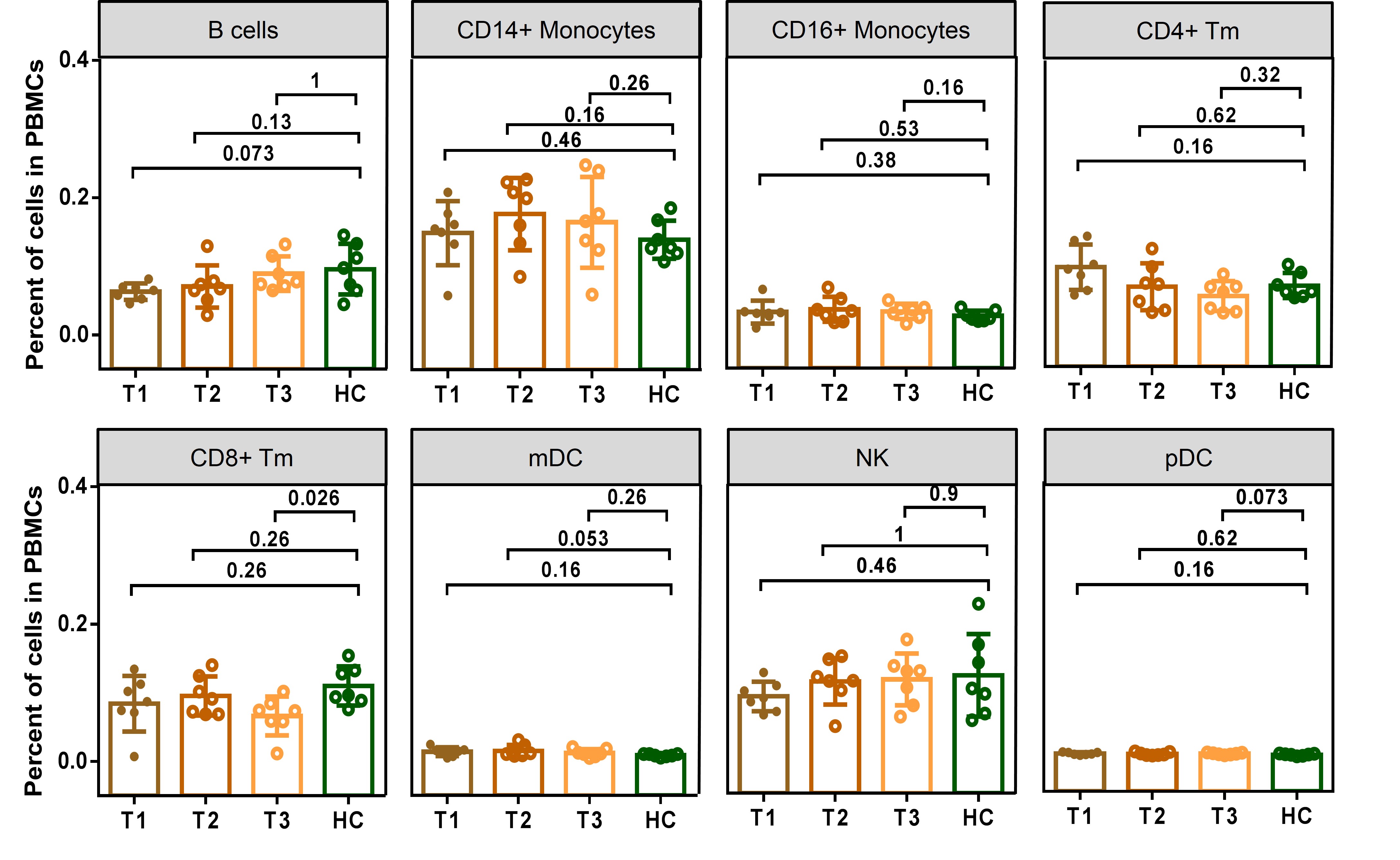


**Figure S4. Comparison of cell subtypes proportions across conditions.** Cell proportions comparison of major cell types between controls and all MDD patients across conditions.


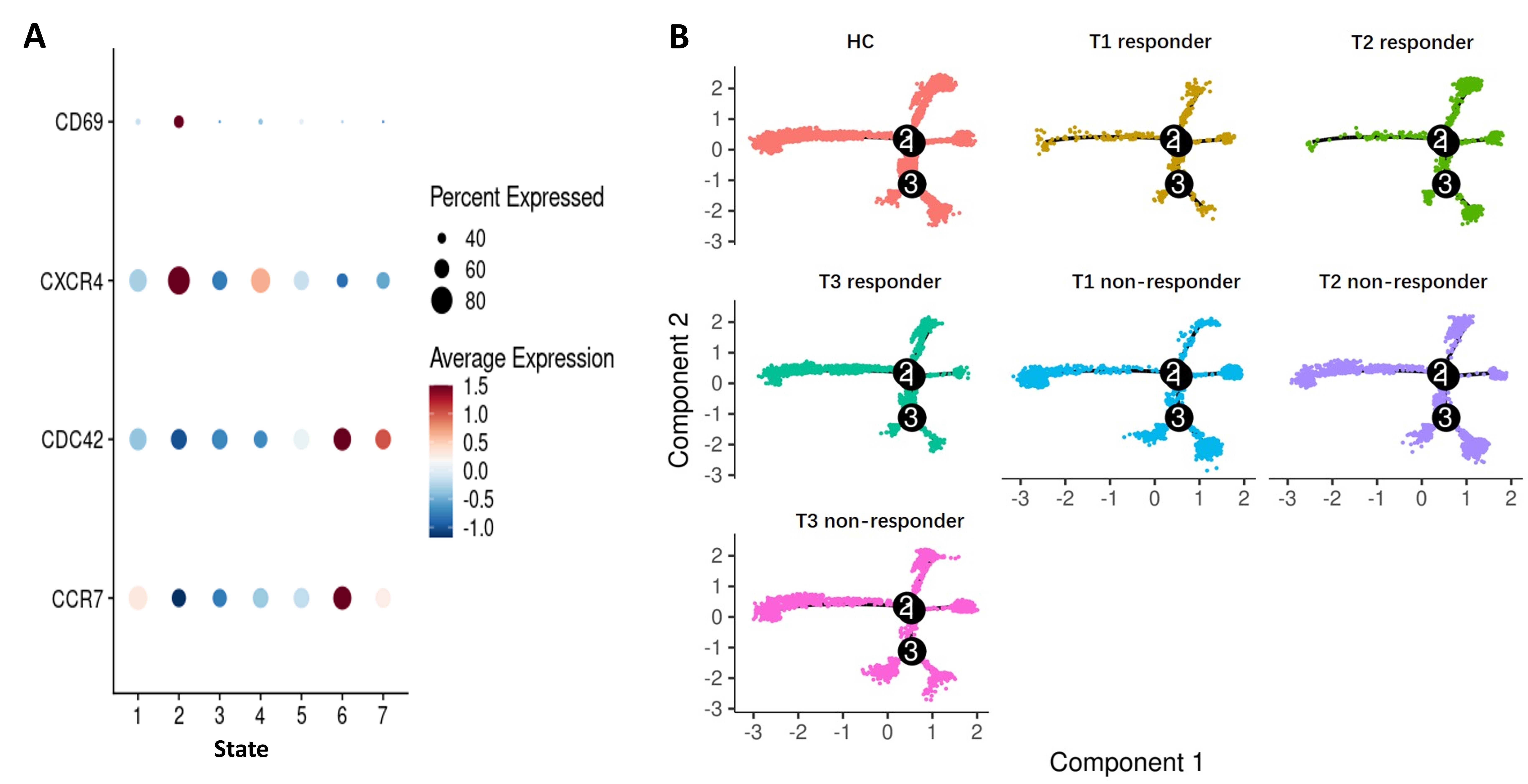


**Figure S5.** The marker genes of states in CD4^+^ naive T cells (A). The differentiation trajectory of CD4^+^ naive T cells, coloured-coded across conditions (B).


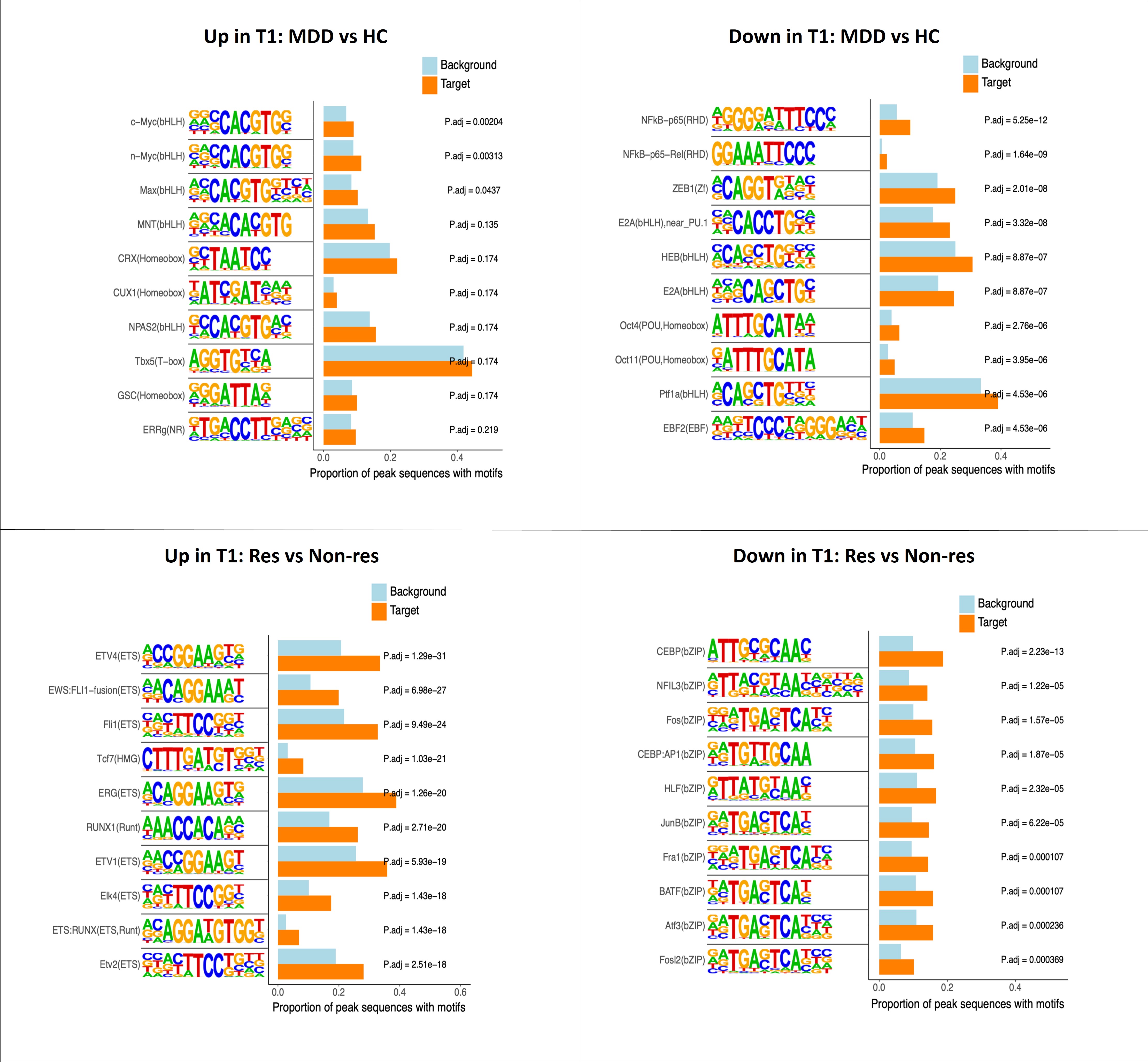


**Figure S6.** Enrichment of known transcription factor motifs in differentially accessible peaks between MDD patients and controls.


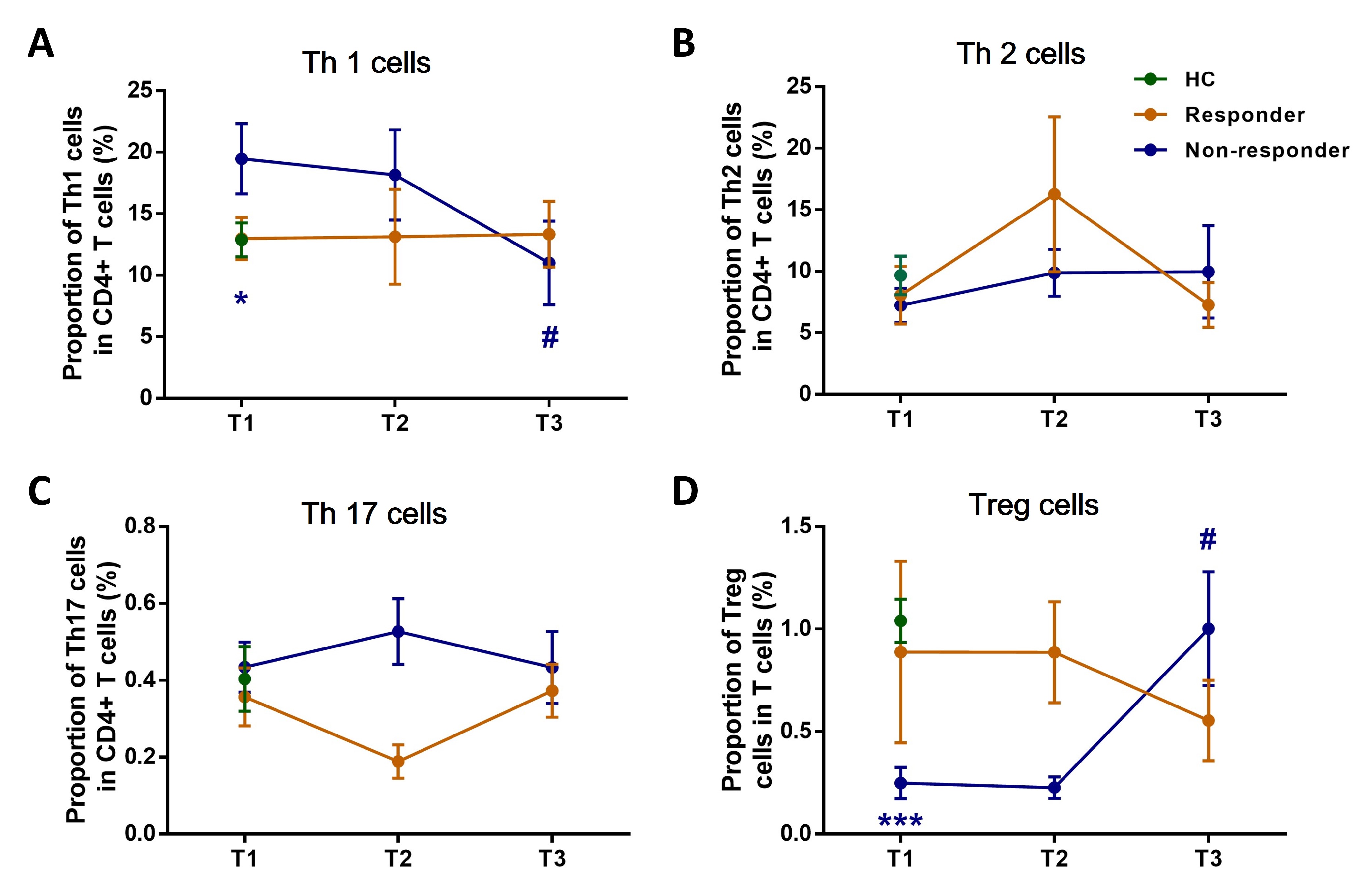


**Figure S7. FACS comparison of helper T cells (Th) and regulatory T cells (Treg) between healthy and MDD patients with different therapeutic outcomes.** A, Th1 cells; B, Th2 cells; C, Th17 cells; D, Treg; E, B cells. The statistical chart is expressed by mean and SD. The treatment responders were defined as the reduction rate of HAMD-17 scores ≥ 50% after 8-12 weeks of treatment compared to their baseline scores. MDD patients who failed to meet the score criteria were identified as non-responders. n = 40 in HC group; n = 19 in responder group; n = 14 in non-responder group. **P* < 0.05 vs. HC group; ****P* < 0.001 vs. HC group; ^#^*P* < 0.05 vs. non-responder group at 0 week.

**Supplementary materials**

**Supplementary Tables**

Table S1. Sociodemographic and clinical data of MDD patients and healthy controls enrolled for scRNA-seq test.

Table S2. Marker genes detected in each cell type.

[Please refer to the uploaded excel for detailed information]

Table S3. Differentially Expressed Genes between baseline MDD patients and healthy controls.

[Please refer to the uploaded excel for detailed information]

Table S4. Comparisons of sociodemographic and clinical data in MDD patients and healthy controls enrolled for flow cytometry test.

**[Tables below]**

**Table S1.** Sociodemographic and clinical data of MDD patients and healthy controls enrolled for scRNA-seq test.

|  | **Age**  **(years)** | **Sex** | **BMI** | **Education** | **Smoking** | **Family**  **history** | **Age of**  **onset (years)** | **Number**  **of**  **episodes** | **Before treatment (0 W)** | | | |  | **After treatment (12 W)** | | | | **Response** |
| --- | --- | --- | --- | --- | --- | --- | --- | --- | --- | --- | --- | --- | --- | --- | --- | --- | --- | --- |
|  |  |  |  |  |  |  |  |  | **HAMD-17** | **QIDS-16** | **YMRS** | **PHQ-9** |  | **HAMD-17** | **QIDS-16** | **YMRS** | **PHQ-9** |  |
| **Patient 1** | 34 | F | 21.26 | U | No | No | 30 | 3 | 17 | 20 | 3 | 14 |  | 16 | 15 | 0 | 21 | No |
| **Patient 2** | 28 | F | 23.14 | U | No | No | 27 | 1 | 20 | 15 | 3 | 20 |  | 16 | 13 | 2 | 7 | No |
| **Patient 3** | 30 | F | 19.05 | H | No | Yes | 26 | 2 | 16 | 13 | 0 | 14 |  | 2 | 4 | 0 | 4 | Yes |
| **Patient 4** | 29 | F | 21.83 | H | No | Yes | 28 | 1 | 19 | 13 | 4 | 11 |  | 4 | 3 | 0 | 1 | Yes |
| **Patient 5** | 36 | M | 19.82 | G | No | No | 31 | 2 | 20 | 11 | 0 | 14 |  | 1 | 3 | 3 | 2 | Yes |
| **Patient 6** | 25 | M | 27.78 | H | No | No | 24 | 1 | 28 | 14 | 0 | 22 |  | 4 | 7 | 0 | 4 | Yes |
| **Patient 7** | 29 | M | 22.49 | U | No | Yes | 24 | 2 | 20 | 14 | 0 | 18 |  | 11 | 11 | 0 | 6 | No |
| **Patient 8** | 36 | M | 22.86 | U | No | No | 31 | 2 | 15 | 14 | 0 | 16 |  | 14 | 9 | 2 | 10 | No |
| **Control 1** | 30 | M | 25.95 | U | No | No | —— | —— | —— | —— | —— | —— |  | —— | —— | —— | —— | —— |
| **Control 2** | 31 | F | 18.94 | H | No | No | —— | —— | —— | —— | —— | —— |  | —— | —— | —— | —— | —— |
| **Control 3** | 31 | F | 24.79 | H | No | No | —— | —— | —— | —— | —— | —— |  | —— | —— | —— | —— | —— |
| **Control 4** | 32 | F | 20.83 | S | No | No | —— | —— | —— | —— | —— | —— |  | —— | —— | —— | —— | —— |
| **Control 5** | 31 | F | 17.63 | H | No | No | —— | —— | —— | —— | —— | —— |  | —— | —— | —— | —— | —— |
| **Control 6** | 26 | F | 23.07 | U | No | No | —— | —— | —— | —— | —— | —— |  | —— | —— | —— | —— | —— |
| **Control 7** | 27 | M | 27.68 | H | No | No | —— | —— | —— | —— | —— | —— |  | —— | —— | —— | —— | —— |
| **Control 8** | 32 | M | 29.05 | P | No | No | —— | —— | —— | —— | —— | —— |  | —— | —— | —— | —— | —— |

Note: MDD, major depressive disorder; F, female; M, male; BMI, body mass index; HAMD-17, 17-Item Hamilton Depression Rating Scale; QIDS-16, 16-item Quick Inventory of Depressive Symptoms–Self-Report; YMRS, Young Mania Rating Scale; PHQ-9, Patient Health Questionnaire.

Education: P: Primary school; S: Secondary school; H: High school; U: University; G: Graduate student.

**Table S4.** Comparisons of sociodemographic and clinical data in MDD patients and healthy controls enrolled for flow cytometry test.

|  | **HCs** | **MDD patients** | ***t/Z/****χ^2^* | ***P*** |
| --- | --- | --- | --- | --- |
| **Number** | 40 | 35 |  |  |
| **Age (years)** | 28.27 ± 7.20 | 29.14 ± 6.67 | -0.542 | 0.590 |
| **Sex (male/female)** | 19 / 21 | 11 / 24 | 2.009 ^a^ | 0.156 |
| **BMI** | 22.81 ± 3.06 | 22.73 ± 4.21 | 0.096 | 0.924 |
| **Family history (yes/no)** | 0 / 40 | 11 / 24 | 14.732 ^a^ | < 0.001 |
| **First episode (yes/no)** | NA | 20 / 15 | NA | NA |
| **HAMD-17 (0 W)** | NA | 20.89 ± 4.54 | NA | NA |
| **HAMD-17 (2 W)** | NA | 14.55 ± 6.27 | NA | NA |
| **HAMD-17 (12 W)** | NA | 11.63 ± 6.84 | NA | NA |
| **Responder (yes/no)** | NA | 19 / 16 | NA | NA |
| **Proportion of CD4^+^ cells (%)** | 41.72 ± 11.52 | 34.14 ± 14.14 | 2.557 | 0.013 |
| **Proportion of naive CD4^+^ cells (%)** | 17.73 ± 8.77 | 7.55 ± 7.82 | -5.023^b^ | < 0.001 |
| **Proportion of effector CD4^+^ cells (%)** | 4.69 ± 6.03 | 10.11 ± 9.25 | -3.685^b^ | < 0.001 |
| **Proportion of CD8^+^ cells (%)** | 38.45 ± 9.61 | 35.14 ± 13.35 | 1.243 | 0.218 |
| **Proportion of naive CD8^+^ cells (%)** | 18.95 ± 9.26 | 9.67 ± 8.26 | -4.354^b^ | < 0.001 |
| **Proportion of effector CD8^+^ cells (%)** | 3.74 ± 4.69 | 9.64 ± 7.30 | -3.898^b^ | < 0.001 |

Note: values represent mean (S.D.)

MDD, major depressive disorder; HCs, healthy controls; BMI, body mass index; HAMD-17, 17-Item Hamilton Depression Rating Scale.

^a^ χ^2^ test, ^b^ Mann-Whitney U test, others were independent sample *t*-test.
